# The Role of the Neuroprotective Factor Npas4 in Cerebral Ischemia

**DOI:** 10.3390/ijms161226144

**Published:** 2015-12-04

**Authors:** Fong Chan Choy, Thomas S. Klarić, Simon A. Koblar, Martin D. Lewis

**Affiliations:** 1 School of Biological Sciences, The University of Adelaide, Adelaide, SA 5005, Australia; fongchan.choy@adelaide.edu.au (F.C.C.); thomas.klaric@alumni.adelaide.edu.au (T.S.K.); 2 School of Medicine, The University of Adelaide, Adelaide, SA 5005, Australia; simon.koblar@adelaide.edu.au; 3 South Australian Health & Medical Research Institute, North Terrace, Adelaide, SA 5005, Australia

**Keywords:** apoptosis, ischemic stroke, neuroinflammation, neuroprotection, Npas4

## Abstract

Stroke is one of the leading causes of death and adult disability in the world. Although many molecules have been documented to have a neuroprotective effect, the majority of these molecules failed to improve the neurological outcomes for patients with brain ischemia. It has been proposed that neuroprotection alone may, in fact, not be adequate for improving the prognosis of ischemic stroke. Neuroprotectants that can regulate other processes which occur in the brain during ischemia could potentially be targets for the development of effective therapeutic interventions in stroke. Neuronal Per-Arnt-Sim domain protein 4 (Npas4) is an activity-dependent transcription factor whose expression is induced in various brain insults, including cerebral ischemia. It has been shown that Npas4 plays an important role in protecting neurons against many types of neurodegenerative insult. Recently, it was demonstrated that Npas4 indeed has a neuroprotective role in ischemic stroke and that Npas4 might be involved in modulating the cell death pathway and inflammatory response. In this review, we summarize the current knowledge of the roles that Npas4 may play in neuroinflammation and ischemia. Understanding how ischemic lesion size in stroke may be reduced through modulation of Npas4-dependent apoptotic and inflammatory pathways could lead to the development of new stroke therapies.

## 1. Introduction

Stroke is the second leading single cause of death and the most common cause of long-term disability in the adult population worldwide [1]. Ischemic stroke is more prevalent than that of hemorrhagic stroke, accounting for approximately 87% of all strokes [2]. Cerebral ischemia occurs as a result of sudden impairment of local cerebral blood flow, leading to irreversible brain tissue damage within an area known as the infarct core. This unsalvageable region is surrounded by the penumbra, a rim of moderately ischemic tissue (due to blood flow from collateral vessels) that is at risk of infarction. As the penumbral tissue is functionally impaired but structurally intact and viable, damage to neurons within the ischemic penumbra is potentially salvageable by means of reperfusion or therapeutic effort [3]. If left without intervention, the penumbral tissue will be recruited gradually to the infarct core, which will expand with time into a maximal volume, and also become irreversibly damaged.

Npas4, which also has a number of other synonyms such as neuronal transcription factor (Nxf) [4,5] and limbic-enriched PAS domain protein (LE-PAS) [6], is an activity-dependent transcription factor belonging to the basic Helix-Loop-Helix (bHLH)-PAS protein family. Npas4 has been demonstrated to play a role in regulating both inhibitory and excitatory synapse formation in a neuronal cell type-specific manner [7,8,9] and controlling the expression of brain-derived neurotrophic factor (BDNF) [7,10], a neurotrophin that is important for neuronal survival, differentiation and synaptic plasticity [11,12]. Studies have also revealed a functional role for Npas4 in hippocampus- [13,14] and amygdala-dependent [15] learning and memory formation, as well as cognitive and social neurobehavior [16]. Due to its central role in homeostasis of neuronal excitation and inhibition, Npas4 has been implicated in a host of psychiatric conditions such as bipolar disorder [17], autism spectrum disorder [18] and cognition-related disorders [19,20]. More importantly, upregulated Npas4 expression has been reported in various brain insults, including focal and global ischemic stroke [4,21,22,23,24].

Previously, although not in the context of ischemic damage, it has been shown that Npas4 plays a critical role in protecting neurons against neurodegenerative insults [23,24,25]. Recently, our laboratory has demonstrated that Npas4 indeed has a neuroprotective role in ischemic stroke and that Npas4 might be involved in modulating the post-stroke inflammatory response [26]. This review summarizes the current knowledge of the roles that Npas4 may play in stroke and describes the possible cellular and molecular mechanisms by which Npas4 links the neuroinflammatory and ischemic processes.

## 2. Npas4 Is a Member of the Basic Helix-Loop-Helix (bHLH)-PAS Transcription Factor Family

### 2.1. The bHLH-PAS Family of Transcriptional Regulators

The bHLH-PAS proteins are members of the bHLH superfamily of transcription factors that generally contain two structurally conserved PAS domains [27]. Studies have shown that bHLH-PAS proteins are involved in many biological processes such as the physiological responses to hypoxia, metabolism of xenobiotics and central nervous system (CNS) development [28]. For example, hypoxia-inducible factor 1 alpha (Hif-1α), the first identified member of the Hif family, has been shown to promote angiogenesis and erythropoiesis by inducing erythropoietin and vascular endothelial growth factor (VEGF) under hypoxic conditions [29]. Mice deficient in Hif-1α die *in utero* by embryonic Day 10 due to abnormal vascularization [30]. Aryl hydrocarbon receptor (Ahr), one of the best characterized bHLH-PAS proteins, has been demonstrated to respond to environmental pollutants by initiating transcription of xenobiotic response genes [31], while murine single-minded 1 (Sim1), the mammalian homolog of the *Drosophila* Sim protein, has been determined to play a role in CNS development, specifically the development of the hypothalamic-pituitary axis [32].

The bHLH-PAS factors are not active individually but function as homo- or heterodimers and thus, they can be subdivided into two classes according to their partnering behavior. Class I bHLH-PAS factors, which include Ahr, the Hif family (Hif-1α, Hif-2α and Hif-3α) and the Sim proteins (Sim1 and Sim2), neither homodimerize nor heterodimerize with other members of the Class I family. To form transcriptionally active complexes, Class I factors must dimerize with Class II bHLH-PAS factors, which promiscuously heterodimerize with other Class I factors and are also able to form homodimers. The best studied Class II protein is the ubiquitous aryl hydrocarbon receptor nuclear translocator (Arnt) which is the most common dimerization partner for both the Ahr and Hif proteins. Other members of the Class II family include the tissue-restricted Arnt2 and the circadian rhythm proteins brain and muscle Arnt-like 1 (Bmal1) and Bmal2 [28,33]. In general, a functional bHLH-PAS transcriptional complex is formed when a Class I factor dimerizes with its respective partner protein from Class II. The choice of partner protein determines the DNA binding specificity of the dimer for target gene regulatory elements.

By definition, members of the bHLH-PAS family contain several highly conserved domains and thus, they also share a common domain organization. The N-terminal half of these proteins contains a bHLH domain which is used in dimerization with other bHLH-PAS proteins and also forms part of the DNA-binding interface of the resulting dimer [34]. Adjacent to the bHLH domain is the PAS homology domain consisting of two repeats, termed PAS A and PAS B, which are separated by a spacer region of variable length [35]. PAS domains are not confined to the DNA-binding bHLH transcriptional regulators but are found in more than 200 proteins, where they act as sensor domains that respond to diverse stimuli such as oxygen, light, small ligands, and redox potential [36]. When present in bHLH-PAS proteins, the PAS domains determine partner choice and prevent dimerization with inappropriate members of the bHLH-PAS family [33]. In addition to contributing to dimerization specificity, they also confer dimer stability [37] and subsequently, have been shown to have a role in DNA binding [33]. Npas4 is the newest member of the bHLH-PAS family of regulatory proteins [4,5,6]. It is so called because of its expression pattern, which is largely restricted to neurons of the brain, and because it contains a conserved PAS domain.

### 2.2. Structure of the Mouse Npas4 Gene and Its Transcript

**Figure 1:**
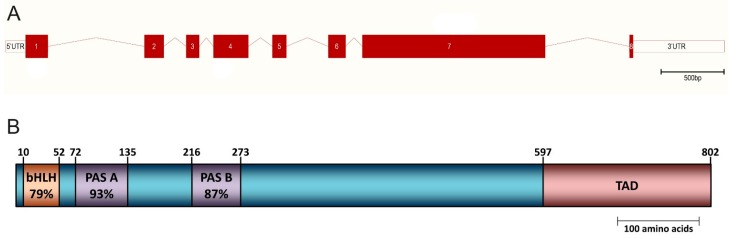
(**A**) The genomic structure of the mouse *Npas4* gene. The *Npas4*-coding region is organized into eight exons and spans a genomic region of approximately 5.6 kb. Filled boxes represent exons, empty boxes represent UTRs and lines between filled boxes represent introns; (**B**) The domain structure of the mouse Npas4 protein showing the location of the basic Helix-Loop-Helix (bHLH), Per-Arnt-Sim (PAS) domains as well as the transactivation domain (TAD). Conservation of each domain to the domain consensus sequence is also indicated (percentage amino acid identity).

In the mouse genome, the *Npas4* gene is located on the reverse strand of chromosome 19A and spans a region of approximately 5.6 kilobases (kb) (Figure 1A). The human *NPAS4* gene is mapped to chromosome 11q13 and its sequence is well conserved among mammals [5]. The total length of the primary mouse *Npas4* transcript is approximately 3277 base pairs (bp) and it consists of a 2409 bp coding region which is flanked by untranslated regions (UTRs) of 155 bp at the 5’ end and 713 bp at the 3’ end. The predicted molecular mass of the mouse Npas4 protein is 87.4 kilodalton (kDa), though *in vitro* transcription and translation of a complementary DNA (cDNA) sequence coding for the full-length mouse Npas4 protein produced a protein of approximately 110 kDa [6].

### 2.3. Domain Structure and Sequence Conservation within the Npas4 Protein

The Npas4 protein consists of 802 amino acids and contains a conserved N-terminal bHLH domain spanning residues 10 to 52 (79% identity with consensus domain sequence) followed by two PAS domains at residues 72 to 135 and 216 to 273 (93% and 87% identity with consensus sequence, respectively) (Figure 1B) [4]. In addition to these conserved bHLH and PAS domains, it has been demonstrated by a reporter gene assay that Npas4 also contains a transactivation domain (TAD) in the C-terminal half of the protein (between residues 597 to 802) [5]. Interestingly, while the TAD was shown to be essential for the transcriptional activation of the reporter gene, Hester *et al.*, observed an increase in activity when the PAS B domain was present, suggesting that this domain is also required for the optimal transcriptional activity of Npas4 [23]. In contrast to the bHLH and PAS domains (which are well conserved among the bHLH-PAS transcription factor family), the overall amino acid sequence of the Npas4 protein has a low degree of homology to other bHLH-PAS factors. Indeed, it shares only 24% overall amino acid identity with Sim2, the protein with which it shares the highest degree of homology [5].

### 2.4. Npas4 and Its Interaction with Other bHLH-PAS Factors

Using a mammalian two-hybrid system, Ooe *et al.*, demonstrated that the NPAS4 protein formed heterodimer complexes with several Class II bHLH-PAS family members, namely ARNT, ARNT2 and BMAL1, but not with BMAL2. Of these proteins, NPAS4’s preferred binding partner is ARNT2 whose expression is known to be abundant and restricted to the brain [38]. In addition, NPAS4 did not homodimerize or heterodimerize with Class I factors such as SIM2 and circadian locomotor output cycles kaput (CLOCK) [5], suggesting that it belongs to Class I of the bHLH-PAS family. A subsequent study by the same group verified these data *in vivo* using a co-immunoprecipitation assay of whole brain extracts to show that the ARNT proteins are the preferred dimerization partners of NPAS4. Although it was observed that the NPAS4 protein exhibited a stronger interaction with ARNT2 than ARNT, the reporter assay showed similar levels of transcriptional activity with NPAS4/ARNT and NPAS4/ARNT2 complexes [24]. Furthermore, it has been shown that while both *Npas4* and *Arnt* mRNAs were detected in the striatum, no significant *Arnt2* expression was observed [39]. Therefore, these data suggest that even though Npas4 has a greater affinity for Arnt2, in some regions of the brain (such as the striatum), Arnt can form functional complexes with Npas4 and could play an independent role in regulating Npas4 signaling *in vivo*.

## 3. Expression of Npas4

### 3.1. Npas4 Expression in Adult Tissues

Expression of bHLH-PAS factors varies from those that are ubiquitously expressed, such as Arnt, to those whose expression is spatially restricted to specific tissues. Like the related bHLH-PAS factor Sim2, Npas4 has a restricted expression pattern and is found in only a very small number of tissues in the adult organism. Northern blot analysis of various rodent tissues revealed that *Npas4* is primarily expressed in the brain where a major transcript of approximately 3.5 kb was detected, though low level expression of additional transcripts (ranging from 4 to 6 kb) was also seen in the testis [6,22,23]. The size of the transcript detected in brain samples corresponds to the expected size of the mature *Npas4* mRNA, however it is unclear whether the larger transcripts present in the testis are true isoforms of *Npas4*. These observations suggest that Npas4, similar to Hif-1α and Bmal2 (which have been shown to be encoded by different tissue-specific mRNA isoforms) [40,41], might undergo alternative splicing in different tissues. In human, the *NPAS4* transcript was mainly expressed in brain tissue, though, unlike in rodents, no expression was observed in the testis [5]. This brain-specific expression pattern seems to be conserved even in lower vertebrates as demonstrated by our recent study which showed that, in the adult zebrafish, the expression of the zebrafish *Npas4* homolog *npas4a* was also restricted to the brain [42].

### 3.2. Enriched Npas4 Expression in the Limbic System of the Brain

In the resting state (*i.e.*, a normal, unstimulated brain), Npas4 is expressed throughout the whole brain at a low level, though it is enriched in certain regions such as the limbic system. The limbic system is loosely defined, in both an anatomical and functional sense, as there is still some controversy regarding precisely which brain structures comprise the limbic system and hence which aspects of neurophysiology it regulates [43]. Most commonly, the limbic system refers to a collection of subcortical structures, including the hippocampus, amygdala, fornix and cingulate gyrus, and it is considered to be involved in learning and memory, regulation of emotion and emotional behavior, sexual function and olfaction [44,45,46].

Using immunohistochemical staining and *in situ* hybridization (ISH), it was shown that both *Npas4* transcript and protein are selectively expressed in the grey matter, but not in the white matter [22]. Of all brain structures, Npas4 is most abundantly expressed in the hippocampus with the highest expression being detected in the pyramidal cell layers of the cornu ammonis (CA1, CA2 and CA3 regions) and the granule cell layer (GCL) of the dentate gyrus (DG) [4,5,6,22]. In addition, more recent studies have also detected the expression of Npas4 protein in other limbic structures such as the lateral nucleus of the amygdala and components of the basal ganglia, for example the nucleus accumbens and ventral pallidum [15,21]. These observations indicate that Npas4 is a neuronal transcription factor whose expression is enriched in the limbic system.

Brain structures outside of the limbic system that have lower levels of Npas4 expression include the cortex, striatum, olfactory bulb (OB), cerebellum and hypothalamus [5,6,21,47,48]. In the cortex, expression is highest in layers III and V and is present in the frontal, parietal and entorhinal cortices [5,6,21,22]. Some species-specific differences in *Npas4* expression have been observed in the cerebellum; in the rat cerebellum, *Npas4* expression is confined to the Purkinje cell layer, while in the mouse cerebellum, *Npas4* is expressed in both the GCL and the Purkinje cell layer [5,6].

### 3.3. Subcellular Localization of Npas4

As a member of the bHLH-PAS family of transcriptional regulators, Npas4 is expected to be localized in the nucleus. This hypothesis was confirmed by an *in vitro* experiment whereby a green fluorescent protein (GFP) construct containing the *Npas4*-coding region was transfected into the COS-7 monkey kidney cells and the fluorescent GFP signal was detected within the nuclei of the transfected cells [6]. This result was in line with the bioinformatic analysis in the same study which predicted a nuclear localization for the Npas4 protein. In addition to the nucleus, it has been shown that the Npas4 protein can be found in other parts of the cell. In rat primary cortical neurons, endogenous Npas4 protein was detected by immunofluorescence in the soma, neurites and also at synapses where it co-localized with the synaptic marker synaptophysin. These data suggest that Npas4 could act as a dendritic transcription factor modulating neuronal activity and might be involved in synaptic plasticity in the brain [22].

### 3.4. Cellular Distribution of Npas4

It has been stated that Npas4 is predominantly expressed in excitatory neurons [7], though subsequent studies have revealed that Npas4 can also be detected in γ-aminobutyric acid (GABA)ergic inhibitory medium spiny neurons in the limbic striatum [21], somatostatin (SST)-expressing interneurons in the medial ganglionic eminence (MGE) at embryonic day 14 (E14) [9] and newborn OB interneurons [49]. Interestingly, there is also evidence that Npas4 is expressed by cells other than neurons. Using immunostaining, Yun *et al.*, reported that, in the subgranular zone (SGZ) of the DG, a small number of Npas4-expressing cells were also found to express markers of neural progenitor cells (NPCs) such as SRY (sex determining region Y)-box 2 (Sox2) and doublecortin (Dcx) [14]. In line with this, using two independent *in vitro* models of neurogenesis, our laboratory has found that while Npas4 is not expressed in undifferentiated embryonic stem cells, it becomes transiently upregulated at a stage that is characterized by proliferation of NPCs [50].

## 4. Regulation of Npas4 Expression by Neuronal Activity

### 4.1. Activity-Dependent Regulation of Npas4 Expression in Neurons

Neuronal activity can be defined as the propagation of a signal from one neuron to another. Such signals are transmitted in the form of neurotransmitter molecules, which are generally described as “excitatory” (such as glutamate) or “inhibitory” (such as GABA), where they bind to specific receptors present on the membrane of the postsynaptic cell and trigger a cascade of events that subsequently lead to the generation of an action potential or changes in the gene expression profile [51,52,53]. Although there are many types of stimuli that can induce Npas4 expression, the primary signal for inducing the expression of Npas4 is an increase in nuclear calcium (Ca^2+^) concentration that, in neurons, is largely regulated by excitatory neuronal activity.

Npas4 expression is rapidly induced (with a peak at approximately 1 h) in response to neuronal activity, characteristic of the group of immediate early genes (IEGs) [7,54,55]. However, this induction is transient as both *Npas4* mRNA and protein have been shown to return to basal levels within 2 to 4 h following peak expression [7,55]. This activity-dependent induction of Npas4 has been demonstrated both *in vitro* and *in vivo* by a number of different methods of stimulating excitatory synaptic activity such as the use of glutamate, which resulted in global activation of glutamate receptors (GluRs) [55] or the use of glutamate analogs *N*-methyl-d-aspartic acid (NMDA) [56,57] and kainate [24], which selectively activate specific GluR subtypes. Predictably, this upregulation of Npas4 expression was inhibited by the treatment with various GluR antagonists such as MK-801 [55], *R*-2-amino-5-phosphonopentanoate (APV) [56] or 6-cyano-7-nitroquinoxaline-2,3-dione (CNQX) [7], suggesting that these ligand-gated ion channels play an essential role in Npas4 signaling.

Recently, it was reported that the formation of neuronal activity-induced DNA double strand breaks (DSBs) in the promoter of *Npas4* was required for its expression [58]. Cultured primary cortical neurons incubated with etoposide, a topoisomerase II (Topo II) inhibitor that causes DNA DSBs [59], for 6 h resulted in an upregulation of 12 neuronal activity-dependent genes, including *Npas4*. Further analysis suggested that this activity-dependent formation of DSBs is likely to be mediated by Topo IIβ as knockdown of Topo IIβ attenuated both DSB formation and *Npas4* expression in response to neuronal stimulation [58].

### 4.2. Nuclear Ca^2+^ Signaling and Regulation of Npas4 Expression

More specifically, it was revealed that the activation of GluRs *per se* is not the determining factor for Npas4 induction but rather the increase in nuclear Ca^2+^ that is a direct consequence of neuronal activity and membrane depolarization. For example, it was demonstrated that Npas4 expression induced by the GABA receptor antagonist bicuculline could be abolished by pretreatment with the Ca^2+^ chelator ethylene glycol tetraacetic acid (EGTA) [7]. Even treatment with potassium chloride (KCl) to induce membrane depolarization (without activating GluRs) was demonstrated to stimulate the expression of Npas4 both *in vitro* [7] and *in vivo* [23]. Indeed, *Npas4* upregulation was observed even in the absence of neuronal activity or membrane depolarization by treating cells with thapsigargin, an inhibitor of the sarco(endo)plasmic reticulum Ca^2+^-ATPase (SERCA) transporter which pumps cytosolic Ca^2+^ into the endoplasmic reticulum. The resultant increase in cytosolic Ca^2+^ concentration was able to induce *Npas4* expression which peaked at 30 min before subsequently declining to baseline levels by 4 h [24]. Finally, inhibition of nuclear Ca^2+^ signaling with calmodulin (CaM) binding-peptide (CaMBP4), a nuclear protein that binds to and inactivates the nuclear Ca^2+^/CaM complex, was demonstrated to prevent the induction of *Npas4* [25].

The exact nature of the molecular events downstream of nuclear Ca^2+^ signaling that lead to Npas4 upregulation are not completely understood, though a number of recent advances are beginning to shed light on the matter. The induction of *Npas4* was also found to be partially reliant on Ca^2+^/CaM-dependent protein kinase type IV (CaMKIV) function [25], suggesting that there may be targets of CaMKIV that regulate *Npas4* transcription. One possible candidate is the cyclic adenosine monophosphate (cAMP) response element-binding protein (CREB), a Ca^2+^-sensitive target of CaMKIV, however the likelihood that the transcription of *Npas4* is regulated by CREB seems improbable given that the promoter region of the *Npas4* gene does not contain any cAMP response element (CRE) sites [25]. On the other hand, there are several downstream regulatory element (DRE) sites present in the *Npas4* proximal promoter, making it likely that Npas4 expression is under the control of DRE-antagonist modulator (DREAM), a transcriptional repressor which is released from DNA upon binding of Ca^2+^ [60]. Indeed, using a chromatin immunoprecipitation (ChIP) assay, it was shown that DREAM bound directly to the *Npas4* promoter and that treatment of neurons with KCl resulted in the Ca^2+^-dependent unbinding of DREAM from the promoter [61].

Given that Ca^2+^ is a ubiquitous second messenger present in all cell types, one might ask what are the factors that drive neuron-specific expression of Npas4? The answer to this question probably lies within the promoter and intron I of *Npas4* gene where putative RE1-silencing transcription factor (REST) binding sites were recently identified using comparative genomics [62]. REST is a transcriptional repressor that is primarily expressed in non-neuronal cells where it silences expression of neuronal genes [63]. Using a reporter assay, it was shown that REST indeed was able to repress *Npas4* transcription in embryonic stem cells, indicating that Npas4 expression is negatively regulated by REST in non-neuronal cells [64].

## 5. Npas4 Expression in Response to Cerebral Ischemia

Cerebral ischemia is a condition in which blood flow to the brain is not sufficient to meet metabolic demand. This rapidly leads to energy depletion within neurons as adenosine triphosphate (ATP) production by oxidative phosphorylation ceases. Without energy, ATP-dependent ion pumps and neurotransmitter transporters fail which leads to loss of membrane potential and uncontrolled glutamate release into the extracellular space [65]. Both of these events trigger repetitive peri-infarct depolarization of neurons within the penumbra leading to increased levels of intracellular Ca^2+^ and induction of activity-regulated genes such as *Npas4*. Ischemic gene expression can activate both cell survival programs and pathogenic cascades, including pro-inflammatory, apoptotic and/or necrotic pathways [66].

A microarray study of gene expression following stroke showed that *Npas4* was one of the most profoundly upregulated genes in the rat brain following 2 h of middle cerebral artery occlusion (MCAo). Temporal profiling revealed that *Npas4* mRNA induction exhibited a biphasic pattern, with an early upregulation at 0.75 h of reperfusion before decreasing to baseline levels between 3 to 6 h and a subsequent transient induction at 12 h before once more returning to basal levels. Using ISH, it was shown that the *Npas4* transcript was elevated throughout the entire challenged hemisphere in both the ischemic core and penumbra, with no change being observed in the contralateral (unchallenged) hemisphere. In the peri-infarct regions, such as cingulate cortex and medial striatum, *Npas4* mRNA induction was high but transient and the transcript returned to control levels after 3 h of reperfusion. On the other hand, the induction of *Npas4* in the ischemic core was moderate but persisted for a period of up to 12 h after reperfusion. Analysis of Npas4 protein expression after focal ischemia using Western blot has shown that no upregulation of Npas4 was detected beyond 3 h of reperfusion [22].

Our laboratory has shown that there was a robust but transient induction of Npas4 protein that peaked 1.5 h following the induction of stroke before progressively declining to baseline levels by 12 h post-stroke. In addition, this stroke-induced upregulation of Npas4 was found not only in the ischemic penumbra but also detected in brain regions outside of the penumbra, more specifically in the corticolimbic system that is critically linked to emotion and cognition [21]. Recently, using oxygen and glucose deprivation (OGD) as an *in vitro* model of ischemia, we also found that *Npas4* transcript levels were upregulated in primary mouse cortical neurons [26].

Global cerebral ischemia, caused by clamping of the common carotid arteries for 10 min, produced a much more sustained response in *Npas4* mRNA expression. Upregulation of *Npas4* was most evident in the hippocampus where increased expression was observed for up to 24 h after reperfusion [22]. Furthermore, the increase in *Npas4* expression was directly correlated with the duration of the ischemic insult and hence, the severity of neuronal damage [22]. Expression of Npas4 protein was detected in surviving cells within the CA1 and CA2 regions of the rat hippocampus 10 days after transient global cerebral ischemia [24] and likewise, in the cortex and hippocampus (CA1 and CA3 regions) after chronic global ischemia [55].

## 6. Functions of Npas4 Following Ischemic Brain Injury

### 6.1. Neuroprotection

It is well established that neuronal survival is dependent on physiological levels of synaptic activity as blockade of normal excitation *in vitro* or *in vivo* causes cell death [67,68]. Activity-dependent neuroprotection is induced by Ca^2+^ entry specifically through synaptic NMDA receptors (NMDARs) [57] and requires the propagation of Ca^2+^ transients to the nucleus [52,69,70]. Interestingly, *Npas4* expression has also been shown to be induced specifically by activation of synaptic, but not extrasynaptic, NMDARs [57]. More importantly, there is increasing evidence which suggests that Npas4 also has a role in this activity-dependent neuroprotective response.

The neuroprotective effect of Npas4 has been demonstrated in various animal models of acute neurological injury. Using a rat model of seizure, Zhang *et al.*, showed that dorsal hippocampal neurons transduced to express Npas4 using an adeno-associated virus (AAV) vector were protected against kainic acid (KA)-induced cell death with a 92% inhibition of cell death compared to the non-injected side (contralateral). Conversely, widespread KA-induced neuronal cell death was observed in animals injected with negative control vectors (*i.e.*, *LacZ* and Empty), suggesting that Npas4 protects against seizure-induced cell death [25]. Using a different approach, Ooe *et al.*, generated a knockout mouse model for Npas4 to understand its functions *in vivo*. They were able to show that Npas4 knockout (Npas4^−/−^) mice were morphologically indistinguishable from their wild-type littermates, with no growth retardation and no significant abnormality. However, they had a shorter lifespan and by 16 months, only about 20% to 30% of Npas4^−/−^ mice survived while almost all wild-type mice survived. Intriguingly, the brains of Npas4^−/−^ mice showed signs of cumulative neurodegeneration such as a significant increase in glial fibrillary acidic protein (GFAP) expression when compared with wild-type littermates [24], which is an indication of glial cell activation and thus neuronal damage [71]. In addition, when challenged with a dose of kainate that was not fatal for the wild-type littermates, three out of eight Npas4^−/−^ mice died within five days and the remaining mice exhibited serious abnormal behavior and severe brain damage [24].

Npas4 has also been linked to the neuroprotective phenomenon of preconditioning. Cortical spreading depression (CSD), which is characterized by slowly propagating waves of depolarizing activity, is a form of pre-ischemic conditioning that has been shown to mediate neuroprotection against subsequent cerebral ischemia [72,73]. Interestingly, it has been shown that both *Npas4* mRNA and protein were transiently induced in neuronal cells following CSD [23]. Further analysis demonstrated that Npas4 was necessary for the protective effect of CSD since F-11 cells (a fusion product of embryonic rat dorsal root ganglion (DRG) cells with a mouse neuroblastoma cell line N18TG-2) treated with RNA interference (RNAi) against Npas4 were shown to be more susceptible to potassium cyanide (KCN)-induced cell death [23]. In a similar study, pretreatment of mouse hippocampal neurons with bicuculline for a period of 12 to 16 h was shown to reduce the percentage of cells that underwent apoptosis following withdrawal of growth factors or treatment with the apoptosis-inducing agent staurosporine. Importantly, the neuroprotective effect of bicuculline pretreatment was abolished when neurons were infected with an Npas4-RNAi AAV construct [25].

Recently, the neuroprotective role of Npas4 has also been extended to ischemic damage. Using OGD, our laboratory has demonstrated that the knockdown of Npas4 in cultured neurons resulted in increased susceptibility to cell death as demonstrated by the increased propidium iodide membrane permeability. The protective effect of Npas4 was further investigated *in vivo* using a photochemically-induced stroke model in mice. We found a significantly larger lesion size and increased neurodegeneration in Npas4^−/−^ mice when compared with wild-type mice following the induction of cerebral ischemia, confirming that Npas4 is neuroprotective in ischemic stroke [26].

The central role of BDNF in neuronal survival is well documented [74,75,76] and there is some evidence that Npas4 regulates the activity-dependent transcription of *BDNF*. In a DNA microarray experiment performed in E16 mouse hippocampal neurons, it was shown that *BDNF* expression was reduced by approximately two-fold in primary neuron cultures with knockdown of Npas4 expression using RNAi. Moreover, cultured neurons from mice deficient in Npas4 exhibited a reduction in KCl-induced *BDNF* mRNA expression as compared to wild-type controls. Using a ChIP assay, the Npas4 protein was shown to bind to *BDNF* promoters I and IV with approximately equal affinity, but not to the coding region or 3′ UTR, in primary rat cortical cultures treated with KCl [7]. A similar result was reproduced in a non-neuronal human cell line; when both NPAS4 and ARNT2 were co-transfected into HEK 293 cells, the subsequent ChIP experiment demonstrated that NPAS4-ARNT2 dimer was enriched relatively more on human *BDNF* promoter I than on promoter IV [10].

### 6.2. Apoptosis

Surprisingly, while it was shown that Npas4 expression enhances cell survival and is neuroprotective [23,24,25,26], Hester *et al.*, reported that AAV-mediated overexpression of Npas4 in cultured cells led to a decrease in cell viability over several days of culture compared to cells infected with a control vector expressing GFP. More specifically, this loss of viability was lessened by removal of the Npas4 C-terminal TAD, suggesting that Npas4-dependent transcriptional activity is essential for the observed cytotoxic effect. Using microarray analysis with HeLa cells overexpressing Npas4, it was shown that the pro-apoptotic gene *Bax* was upregulated and this result was confirmed by Western blot analysis, where a significant increase of Bax protein expression was observed. Subsequently, a ChIP assay was used to demonstrate that the endogenous *BAX* promoter was bound by Npas4 when both Npas4 and Arnt2 were overexpressed in HeLa cells [23]. In line with this study, our laboratory has also revealed that Npas4 plays a role in modulating apoptosis. Npas4^−/−^ mice subjected to focal cerebral ischemia showed a significant decrease in the number of apoptotic cells in the lesion core as compared to wild-type mice as seen by a reduction in both TUNEL staining and the number of cells expressing apoptosis-inducing factor (AIF) [26]. Since the total number of degenerating cells at the lesion site was in fact increased in Npas4^−/−^ mice, we hypothesize that a switch from apoptosis to necrosis may explain this observation as it has been shown that blockade of a specific cell death mechanism does not preclude cell destruction but instead promotes an alternative pathway [77]. It is known that a switch from apoptotic to necrotic cell death is detrimental as the latter is always a pathological process that augments inflammation whereas the former induces anergy or an immunosuppressive phenotype [78]. Therefore, such a switch may also account for the larger lesion size seen in Npas4^−/−^ animals. These findings suggest that one of the protective effects of Npas4 is to limit tissue damage through modulation of the cell death pathway by directing damaged cells to undergo apoptosis instead of necrosis.

### 6.3. Neuroinflammation

In the brain, neuroinflammation is an important defense mechanism that helps to protect the CNS. On the other hand, uncontrolled or extended neuroinflammation is harmful and can induce rapid neuronal death in the ischemic core which progressively expands toward the penumbral area [79]. Interestingly, our laboratory has also shown that Npas4 deficiency resulted in a significant increase in the number of activated microglia and astrocytes 96 h after the induction of stroke [26]. Microglia are the resident macrophages of the CNS [80] and function as scavenger cells in the event of brain injury [81]. Following activation by ischemia, microglia can undergo morphological transformation into phagocytes capable of releasing a variety of cytotoxic and/or cytoprotective substances [82].

Although it is unclear whether microglial activation is necessarily destructive following ischemic stroke, there is a considerable amount of data suggesting a role for activated microglia in worsening brain injury. Treatment with edaravone, a potent free radical scavenger, after focal ischemia led to a marked reduction of brain infarct size and improvement of neurological recovery in mice by reducing microglial activation [83]. Using a rat model of permanent MCAo, Gunther *et al.* demonstrated that repetitive hyperbaric oxygen treatment-induced suppression of microglial activation was able to reduce the infarct volume [84]. Direct evidence has also been presented to support a damaging role of microglia on neuronal cells following ischemic insults when increased neuronal loss was observed in the presence of microglia [85,86]. Conversely, some studies have reported that microglia or their secreted products may in fact protect cells. The protective effects of microglia have been shown to be mediated by their ability to phagocytose infiltrating neutrophils, which are known to secrete free radicals and other inflammatory mediators, and through the elimination of excitotoxins from the extracellular space after stroke [87]. In addition, it has been shown that microglia can synthesize and release several compounds, such as growth factors and neurotrophins, that promote neuronal survival and brain tissue repair in the event of brain injury [88,89].

It is well known that astrocytes carry out a variety of functions, including scavenging free radicals, prevention of excitotoxicity and maintenance of ionic homeostasis, which are critical for normal brain development and function [90]. After brain ischemia, astrocytes are activated resulting in increased production of intermediate filament proteins, such as vimentin and GFAP, and a phenomenon termed reactive astrogliosis occurs, which is characterized by profound morphological and functional changes in astrocytes [91]. It has been demonstrated that astrocytes, like microglia, also participate in post-ischemic brain inflammation by secreting cytokines and inducible nitric oxide synthase (iNOS), as well as factors that can induce additional microglial activation [92]. Astrocytes, together with neurons and endothelial cells, also produce tumor necrosis factor-like weak inducer of apoptosis (TWEAK) that, by interacting with Fn14 receptors present on astrocytes, can stimulate the production of pro-inflammatory molecules [93,94]. Intriguingly, it has been reported that the expression of TWEAK and Fn14 were elevated in a murine stroke model and that intracerebroventricular injection of a soluble decoy (osteoprotegrin (OPG)-Fc protein) to Fn14 significantly reduced the infarct size [94].

Upregulated cytokine expression in the brain has been documented after a variety of insults such as stroke. In addition to being expressed by the immune cells, cytokines are also produced by resident brain cells, including neurons and glial cells [95]. In ischemic brain injury, interleukin (IL)-6 and tumor necrosis factor alpha (TNF-α) are largely thought to act as pro-inflammatory cytokines which exacerbate brain damage and are involved in the initiation of early inflammation [96]. We have noted that Npas4^−/−^ mice had both increased IL-6 and TNF-α expression post-stroke [26]. Remarkably, it has been documented that IL-6 also has an anti-apoptotic function after focal ischemia [97]. Given the fact that we have shown that Npas4 is involved in the caspase-independent apoptotic pathway and that other studies have demonstrated that TNF-α can induce necrosis in cells that failed to activate the pro-survival signaling cascades and apoptosis [98], we hypothesize that the switch from apoptosis to necrosis is responsible for the aggravated brain injury observed in mice lacking Npas4.

Several Npas4 target genes have also been implicated in the inflammatory response. For example, the IEG transcription factor c-Fos, whose expression is regulated by Npas4 [13], belongs to the Fos family of proteins that dimerize with c-Jun to form the activator protein 1 (AP-1) transcription complex [99,100]. Studies have shown that c-Fos plays critical roles in the molecular mechanisms underlying a number of cellular processes, including proliferation, differentiation and apoptosis [101,102]. Interestingly, it has been reported that c-Fos also functions as an anti-inflammatory transcription factor by suppressing the activation of nuclear factor (NF)-κB [103], a transcription factor that regulates the expression of TNF-α and other pro-inflammatory cytokines [104]. Furthermore, a recently identified Npas4 target gene murine double minute 2 (MDM2), an E3 ubiquitin ligase which was upregulated in Npas4^−/−^ OB granule cells [49], has been shown to act as a co-factor for NF-κB at target gene promoters [105]. These findings further support a role for Npas4 in inflammation.

An increasing number of studies have shown that M1/M2 polarization of microglia plays a crucial role in the balance of inflammation [106]. Classically activated microglia (M1 phenotype) are cytotoxic due to the production of reactive oxygen/nitrogen species (ROS/RNS) and pro-inflammatory cytokines that contribute to the inflammatory response and propagation of cell death beyond the initial ischemic region. In contrast, alternatively activated microglia (M2 phenotype) block pro-inflammatory responses by expressing cytokines and receptors that are involved in the inhibition of inflammation and restoration of homeostasis [107]. Given the fact that it has been demonstrated that a microenvironment dominated by pro-inflammatory cytokines favors polarization to M1 microglia and prevents an M2 switch [108] and that ablation of Npas4 resulted in increased IL-6 and TNF-α cytokine levels in the brain following ischemia [26], it is possible that microglia could be primed toward the M1 state in Npas4^−/−^ mice which maybe the underlying cause of the increased stroke-induced brain injury and inflammation observed in these animals.

## 7. Conclusions

Npas4 is an activity-dependent transcription factor whose expression is robustly and transiently upregulated in the brain during the acute phase of ischemia [21,22]. Although previous studies have demonstrated a role for Npas4 in the protection of neurons against neurodegenerative insults, we recently showed the neuroprotective effect of Npas4 in the context of ischemic damage as Npas4 deficiency increased the susceptibility of cultured neurons to OGD-induced cell death and exacerbated the severity of brain injury after focal ischemia in mice [26]. Stroke is one of the leading causes of death and adult disability worldwide. While preclinical studies have demonstrated many molecules as potential neuroprotective factors, the large majority of these molecules were unable to reverse the neurological deficits in ischemic stroke patients [109]. This suggests that factors with a neuroprotective effect alone are not sufficient to improve the prognosis of cerebral ischemia and that neuroprotectants that can regulate other processes which occur in the brain during stroke, such as neuroinflammation and cell death, could potentially be targets for the development of effective therapeutic interventions in stroke.

After stroke, an inflammatory response is initiated within a few hours and is characterized by the activation of astrocytes and microglia, as well as the subsequent infiltration of granulocytes (neutrophils) and monocytes/macrophages [110]. Neuroinflammation is one of the key pathophysiological mechanisms contributing to the progression of brain damage caused by ischemia [95] and inhibition of this response has been shown to decrease infarct size and improve neurological function in animal stroke models [111,112]. Although many experimental anti-inflammatory approaches have been developed and proven successful [113,114,115], attempts to translate this into clinical application have been disappointing [116,117]. This is not surprising since increasing evidence demonstrates that the inflammatory response not only aggravates secondary brain damage in the acute stage of stroke but also beneficially contributes to brain tissue repairing and remodeling after an ischemic event. Therefore, inhibiting inflammation could be detrimental and worsen long-term functional consequences after cerebral ischemia [118,119].

**Figure 2:**
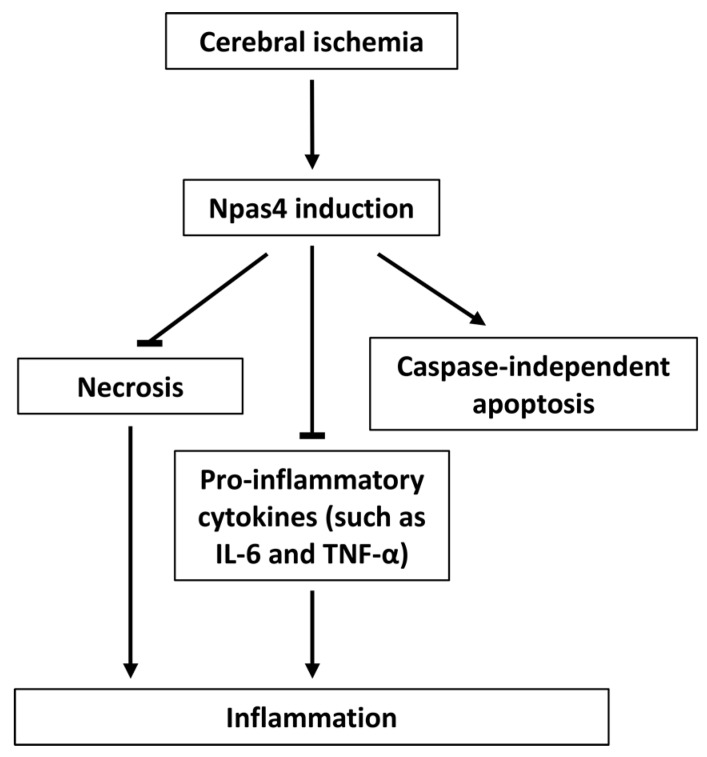
Model of Npas4 neuroprotective function following cerebral ischemia. Ischemic challenge induces Npas4 expression, which then limits brain damage via two mechanisms: (1) by modulating the cell death pathway such that damaged cells undergo apoptosis in preference to necrosis and (2) by suppressing inflammation.

We recently demonstrated for the first time that Npas4 may have the capacity to regulate the brain’s inflammatory response to stroke since genetic ablation of Npas4 significantly increased activated astrocyte and microglial cell numbers, pro-inflammatory cytokines IL-6 and TNF-α levels and caused a switch from apoptotic to necrotic cell death in mice subjected to photochemical stroke [26]. Apoptosis is a highly controlled and regulated form of cell death that is characterized by nuclear condensation, cell shrinkage, membrane blebbing and DNA fragmentation, whereby a cell dies without causing damage to neighboring cells [120]. In contrast, necrosis is a pathological process that results in cellular swelling, disruption of ionic and internal homeostasis, plasma membrane rupture and cell lysis. This swiftly leads to the release of intracellular contents that can contribute to excessive inflammation, edema and collateral damage to the surrounding cells [78]. Therefore, harnessing the multidimensional neuroprotective capacity of Npas4 could limit progressive neurodegeneration and neuroinflammation (Figure 2), potentially providing new strategies for the treatment of ischemic stroke.
